# Seroprevalence and risk factors on Syphilis among blood donors in Chengdu, China,from 2005 to 2017

**DOI:** 10.1186/s12879-019-4128-7

**Published:** 2019-06-10

**Authors:** Shuangli Liu, Liping Luo, Guangxiang Xi, Like Wan, Li Zhong, Xue Chen, Tianxiang Gong, Shuping Li, Yi He, Na Li

**Affiliations:** 1 Department of Blood Collection, Chengdu Blood Center, Chengdu, China; 2 Yibin Blood station, Yibin, China; 3 Department of Blood Supply, Chengdu Blood Center, Chengdu, China; 4 Department of Blood Preparation, Chengdu Blood Center, Chengdu, China; 5 Department of Donor Service, Chengdu Blood Center, Chengdu, China; 6 Blood Screening Laboratory, Chengdu Blood Center, Chengdu, China; 7 Blood research laboratory, Chengdu Blood Center, Chengdu, China; 8 Department of Quality Control, Chengdu Blood Center, Chengdu, China

**Keywords:** Syphilis, Risk factors, Blood safety, Health consultation, Blood donors

## Abstract

**Background:**

High-risk population of blood donation increases the prevalence of transmit blood-borne diseases and harm the blood safety. Syphilis accounts for approximately 10% of commonly sexually transmitted diseases. The risk factors for blood donors infected with syphilis are also risk factors for other blood borne diseases. The objective of the study is to investigate the seroprevalence and risk factors on syphilis among blood donors, and analyze the donation status of high-risk population.

**Methods:**

A retrospective study was conducted in Chengdu Blood Center during 2005 and 2017. Serological test results of volunteer blood donors were collected. Conditional logistic regression models were performed to investigate syphilis-related risk factors and population attributable risk (PAR) was performed to predict the tendencies of high-risk populations’ on risky behaviors.

**Results:**

The serological epidemic for syphilis among blood donors in Chengdu showed an upward trend from 2005 to 2017.TP positive blood donors were more likely to have multiple sexual partners and commercial sex (50.6% vs.22.6, 11.1% vs.4.6%). Multiple condition logistic regression model denoted the following risk factors for increasing rates of syphilis infections: multiple sexual partners (OR = 7.1, 95% CI:1.72–6.58), razor reuse (OR = 1.7;, 95% CI:1.01–2.01); ear piercing (OR = 2.7, 95% CI:1.48–3.37); tattoo (OR = 3.3, 95% CI:1.17–6.78); condom occasionally (OR = 2.8, 95% CI:0.68–1.66). The PAR for each of the risk factors were 0.225, 0.144, 0.147, 0.018, 0.129, 0.018, respectively.

**Conclusion:**

Health consultation and screening of high-risk groups before blood donation need to be further improved. Blood donor recruitment should emphasize on excluding the high-risk donors and recruiting more low-risk blood donors. In addition, this study also shows that sharing cosmetic surgical instrument has been proven to transmit blood-borne diseases. Therefore, the syphilis in blood circulation should not be ignored.

**Electronic supplementary material:**

The online version of this article (10.1186/s12879-019-4128-7) contains supplementary material, which is available to authorized users.

## Background

Blood donation is an important procedure that saves millions of lives. However, unsafe transfusion practices carry the risk of transfusion-transmissible infections (TTIs). An unsafe blood transfusion is very costly from both an economic and a human point of view, not only for the recipients themselves, but also for their families and their communities [1, 2]. In China, hepatitis B virus (HBV), hepatitis C virus (HCV), human immunodeficiency virus (HIV) and Treponema pallidum (TP) in all donations must be underwent routine laboratory testing. Syphilis is a sexually transmitted disease (STD), caused by the spirochete *Treponema pallidum* subsp. *pallidum* (hereafter *Treponema pallidum*), is a chronic, sexually transmitted infection affecting an estimated 36 million people worldwide, with 11 million new cases occurring annually [3]. Syphilis is a multistage disease punctuated by asymptomatic periods of latency. The primary and secondary stages of syphilis present with a painless chancre at the initial site of infection followed by a non-pruritic rash, respectively, both of which spontaneously resolve [4]. The *World Health Organization* (WHO) has conducted a survey on the prevalence of four sexually transmitted diseases: chlamydia trachomatis, *Neisseria gonorrhoeae*, syphilis, and trichomonas vaginalis. It was found that syphilis accounts for approximately 10% of these sexually transmitted diseases [5, 6]. According to the Chinese Health Statistical Digest by the Chinese Ministry of Health (MOH), the incidence of syphilis is second only to viral hepatitis and tuberculosis in chinese class A and B communicable diseases [7].

*Treponema pallidum* can survive for several years at − 78 °C, in the blood from syphilis patients may still be infectious within 4 days storage at − 4 °C [8, 9]. Syphilis and HIV affect similar patient groups and co-infection is common. Infection with syphilis is a risk factor for infection with HIV, HBV, and HCV [10–12]. The risk factors for blood donors infected with syphilis are also risk factors for other blood borne diseases [13–15]. Screening for high-risk groups before blood donation currently depends entirely on pre-donation health consultation. They donate blood or need postpone and withdraw from blood donation depend to the report of blood donors on medical history and dangerous behavior [16, 17]. In fact, many blood donors did not earnestly fill out the “health status inquiry form of blood donors”, some of them did not understand the contents of the questionnaire and could not accurately fill out, or concerned about the privacy disclosure in the process of blood donation on a public environment. Moreover, the diversity of blood donation motives also made it impossible for some blood donors to report truthfully, resulting in some high-risk groups entering the blood donation process. For instance, some blood donors may not know that their behaviors are dangerous behaviors which are susceptible to transfusion diseases, or some blood donors who have the clear risky behaviors intentionally concealed to detect whether they are infected. In addition, some blood donors have not read the health checklist carefully in order to save time.

Blood donation in high-risk groups is a threat to blood safety. It is a matter of concern whether the high-risk group of blood donors is effectively excluded from the health consultation before blood donation. In order to optimize donor selection, a validated donor questionnaire should be used and confidentiality in all steps of donation. The possibility of a confidential self-exclusion should be explicitly pointed out to donors. In this study, we conducted a survey on the seroprevalence and risk factors on syphilis among blood donors in Chengdu from 2005 to 2017.

## Methods

### Study subjects

The blood samples were collected from blood donors in Chengdu from January 2005 to December 2017. Donors with seropositivity of syphilis alone were selected as cases. Controls were matched to cases to control confounding. For each positive case, two syphilis-negative, age- and sex-matched donors were selected as controls. Between January 2005 and December 2017, 368 positive cases and 736 controls were included in the prevalence study. This study was approved by the Medical Ethics Committee of Sichuan University (NO:2014015–02).

## Methods

### Laboratory tests

Blood specimens from the blood donors were tested for HBsAg, anti-HCV, anti-HIV (types 1 and 2), syphilis, and ALT according to procedures stipulated in the Chengdu blood center. The equipment of anti-TP testing: FAME24/20 automatic enzyme immunoassay system (Hamilton, USA). Reagents: anti-TP diagnostic kit (Beijing Wantai, lot number N20110405, N20110708; Beijing Huada love, lot number 20110616, 20110915). Blood specimens were carried out using two different test kits that were approved by the Chinese Food and Drug Administration. Each donation was screened by two ELISA kits regardless of the result of the first one. Each initial reactive sample was further tested in duplicate, according to the manufacturer’s instructions. If two out of three results were positive (Repeat Reactive), the unit was discarded and the sample was classified as positive.

### Subject questionnaire and data collection

Donors with anti-TP infection alone were selected as cases. Controls were matched to cases to control confounding. All the selected donors were contacted by telephone obtained from donor registration forms. After explanation of the study aim some blood donors agreed to participate in the study. A risk assessment questionnaire (Additional file 1) was given to every eligible donor in order to investigate relevant information. The main contents of the questionnaire include: the information consisted of facial shaving by sex, age, nationality, occupation, educational level and marital status; related behaviors including sharing razor, tattooing, dental treatment, ear piercing, transfusion, acupuncture, injection, dental treatment, body fluid contact, condom use, paid sex, sexual contact with syphilis patients. The investigation was conducted by professional medical personnel in order to ensure the accuracy of data collection. All the information was confidential.

### Statistical methods

Calculated the rate of TP-positive, seroprevalence and serological incidence based on the test results. The rate of TP-positive refers to the proportion of the total number of syphilis-positive in the total blood donations, that is, the total number of syphilis-positive as a molecule, and the total population of blood donations as the denominator. Seroprevalence was calculated using the total number of syphilis-positive donors as the numerator in the initial blood donation, the total number of initial blood donors at the same period as the denominator. Serological incidence (%): The total number of syphilis-positive in the repeat blood donors in that year was the numerator, all repeat blood donors during the same period was the denominator. Database was established using Epidata3.1. Data were analyzed using SPSS version 22.0. Possible risk factors for screening of meaningful variables were analyzed by using conditional logistic regression and estimates of the odds ratios (ORs) with their corresponding 95% confidence interval (CI). The crude odds ratios (ORs) and 95% CI were estimated by the univariate conditional logistic regression analysis. Potential interactions between razor sharing and tattooing, ear piercing, condom and number of sex partner were assessed. Correlation analyses, such as Spearman rank correlation were also performed for testing collinearity among independent variables. Forward stepwise methods were used to determine which variables significantly contributed to syphilis infection. All of statistical tests were two-sided and a level of *P* < 0.05 was used to indicate statistical significance. The proportion of all the TP-positive cases in the population of the voluntary blood donors attributable to multiple risk factors (population attributable risk, PAR) was estimated by using Bruzzi’s formula [18] to the observed OR.

## Results

### Seroprevalence and serological incidence

**Fig. 1:**
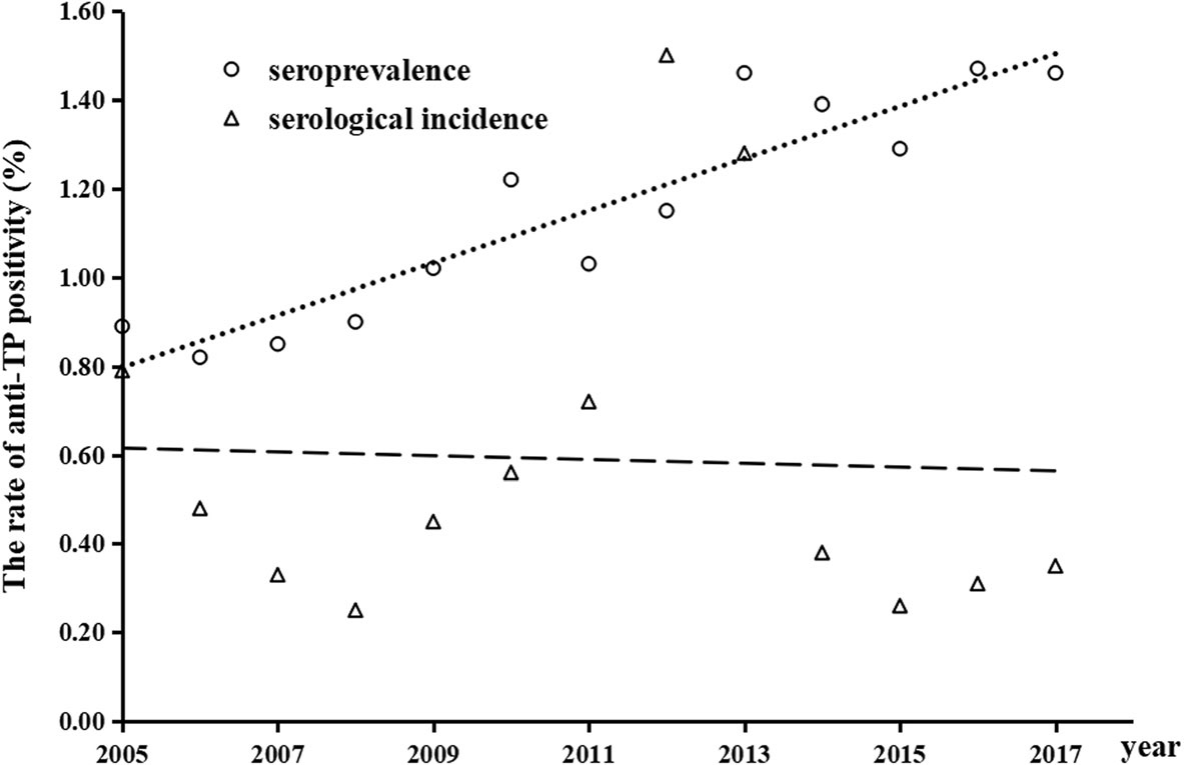
The linearity of seroprevalence and serological incidence in blood donors from 2005 to 2017 (seroprevalence R^2^ = 0.9011, serological incidence R^2^ < 0.9)

A total of 2,100,071 voluntary blood donors were selected in the Chengdu blood center during 2005–2017 for the study. It is worth mentioning that all participants in the study have normal levels of transaminase, and HIV, HBV and HCV tests are negative. Of these, 20,510 blood donations were positive for syphilis (Table 1). The rate of anti-TP positivity was 0.88% corresponding to 815 repeatedly reactive ELISA tests out of 92,610 blood donations in 2005 and the rate of TP-positivity was 0.98 in 2017. The seroprevalence of syphilis in blood donors from 2005 to 2017 in Chengdu also showed an overall upward trend (Fig. 1), while the serological incidence was fluctuated. The seroprevalence of the first-time blood donors in the study period were higher than the serological incidence (Table 1).

**Table 1:** The seroprevalence of syphilis in Chengdu blood center, 2005–2017

	2005	2006	2007	2008	2009	2010	2011	2012	2013	2014	2015	2016	2017
Total number of blood donations	92610	105949	121745	137524	150702	145691	149863	153299	160507	182222	192959	205963	219140
Number of initial blood donors	82304	86948	93272	104551	110174	102357	120561	134350	128455	116679	117722	122212	124779
Number of repeat donors	10306	19001	28473	32973	40528	43334	29302	18949	32052	65543	75237	83751	94361
Total number of syphilis-positive	815	804	889	1019	1302	1486	1452	1825	2280	1869	1709	2052	2157
syphilis-positive in the initial blood donors.	734	712	796	938	1119	1245	1241	1541	1869	1621	1516	1795	1828
syphilis-positive in repeat donors	81	92	93	81	183	241	211	284	411	248	193	257	329
Rate of TP-Positive(%)	0.88	0.76	0.73	0.74	0.86	1.02	0.97	1.19	1.42	1.02	0.89	1.00	0.98
Seroprevalence(%)	0.89	0.82	0.85	0.90	1.02	1.22	1.03	1.15	1.46	1.39	1.29	1.47	1.46
serological incidence (%)	0.79	0.48	0.33	0.25	0.45	0.56	0.72	1.50	1.28	0.38	0.26	0.31	0.35
P value	0.282	<0.001	<0.001	<0.001	<0.001	<0.001	<0.001	<0.001	0.021	<0.001	<0.001	<0.001	<0.001

Seroprevalence(%): the seroprevalence was calculated using the total number of syphilis-positive donors as the numerator in the initial blood donation, the total number of initial blood donors at the same period as the denominator. Serological incidence(%): The total number of syphilis-positive in the repeat blood donors in that year was the numerator, all repeat blood donors during the same period was the denominator

### Risk factors

During the case-control study, 368 (33.3%) of the 1104 eligible anti-TP-positive donors were enrolled in the study. After obtaining informed consent for the study, 368 anti-TP-positive cases and 736 controls were investigated. Reasons for non-response included contact lost and refusing to participate in the study. Overall and subgroup-specific prevalence of *T.pallidum* seropositivity and active syphilis infection were calculated using case definitions described above. The demographic characteristics associated with all samples were shown in Table 2. Demographic variables included marital status, educational attainment, sex, and occupation. In the study, no significant differences were found between the cases and the demographic and socioeconomic characteristics of controls.

**Table 2:** Demographic characteristics of the research subject, 2005–2017

	Case		Control	
	NO.	Percent (%)	NO.	Percent (%)
Marital status				
Single	93	25.3	193	26.2
Married	266	72.3	536	72.8
Divorced	9	2.4	7	1.0
Education				
< Primary school	34	9.2	38	5.2
Middle school	123	33.4	214	29.1
High school	107	29.1	214	29.1
Complete university and above	104	28.3	270	36.7
Sex				
Male	177	48.1	354	48.1
Female	191	51.9	382	51.9
Occupation				
Worker	59	16.0	111	15.1
Farmer	46	12.5	119	16.2
Merchant and commercial service	95	25.8	125	17.0
Government staff	40	10.9	100	13.6
Student	25	6.8	84	11.4
Staff	38	10.3	100	13.6
Other	65	17.7	97	13.2

Note: 1) The people of divorced group included widowed and separated. 2) The other group in occupation included unemployed self-employed and others

By conditional logistic regression, occupation as a risk factor significantly associated with syphilis infection was student, the education was bachelor. Razor reuse, ear piercing, tattoo, dental operation, paid sex, number of sex partner, condom and sexual contact with syphilis (*P* < 0.05; Table 3). After the forward stepwise method was used for variable selection in the multiple conditional logistic regression, interaction terms were checked in the final model. It is shown that no statistically significant interactions were found among these variables: occupation and education. Dental operation and paid sex did not show significant associations with syphilis infection (*P* > 0.05; Table 4).

**Table 3:** The result of univariate logistic regression, 2005–2017

Variable	Case (*n* = 368)	Control (*n* = 736)	β	SE	P	OR	95%CI	
Marital status (control=single)	96 (25.3)	193 (26.2)			0.142			
Married	266 (72.3)	536 (72.8)	0.096	0.222	0.666	1.100	0.713	1.699
Divorced	9 (2.4)	7 (1.0)	1.111	0.568	0.050	3.038	0.998	9.250
Occupation (control=worker)	59 (16.0)	111 (15.1)			<0.001			
Farmer	46 (12.5)	119 (16.2)	-0.321	0.255	0.208	0.725	0.440	1.196
Merchant and commercial	95 (25.8)	125 (17.0)	0.347	0.219	0.113	1.415	0.921	2.175
Government staff	40 (10.9)	100 (13.6)	-0.310	0.263	0.238	0.733	0.438	1.228
Student	25 (6.8)	84 (11.4)	-1.369	0.422	0.001	0.254	0.111	0.582
Staff	38 (10.3)	100 (13.6)	-0.393	0.267	0.142	0.675	0.400	1.140
Others	65 (17.7)	97 (13.2)	0.186	0.239	0.438	1.204	0.753	1.925
Education (control=primary school or less)	34 (9.2)	38 (5.2)			0.001			
Middle school	123 (33.4)	214 (29.1)	-0.510	0.269	0.058	0.601	0.355	1.017
High school	107 (29.1)	214 (29.1)	-0.698	0.280	0.013	0.498	0.288	0.861
> Bachelors	104 (28.3)	270 (36.7)	-1.038	0.288	<0.001	0.354	.201	0.623
Razor reuse	201 (54.6)	332 (45.1)	0.520	0.151	0.001	1.682	1.252	2.261
Ear piercing	129 (35.1)	165 (22.4)	0.997	0.188	<0.001	2.711	1.877	3.916
Tattoo	17 (4.6)	11 (1.5)	1.185	0.400	0.003	3.270	1.492	7.167
Eyebrow tattooing	44 (12.0)	70 (9.5)	.304	0.223	0.174	1.355	0.875	2.100
Dental operation	133 (36.1)	207 (28.1)	0.381	0.139	0.006	1.463	1.114	1.921
Acupuncture	58 (15.8)	88 (12.0)	0.334	0.187	0.074	1.397	0.968	2.016
Transfusion	10 (2.7)	18 (2.4)	0.105	0.394	0.789	1.111	0.513	2.407
Body fluid contact	33 (9.0)	52 (7.1)	0.244	0.228	0.284	0.783	0.501	1.225
Paid sex	41 (11.1)	34 (4.6)	1.067	0.262	<0.001	2.907	1.738	4.861
Number of sex partner (control=0)	24 (6.5)	94 (12.8)			<0.001			
One statilzed sex partner	157 (42.7)	476 (64.7)	0.613	0.324	0.058	1.847	0.979	3.483
≥2	187 (50.8)	166 (22.6)	1.957	0.333	<0.001	7.079	3.685	13.599
Condom (control=always)	84 (22.8)	234 (31.8)			0.001			
occasional	143 (38.9)	231 (31.4)	0.728	0.194	<0.001	2.071	1.414	3.031
never	141 (38.3)	271 (36.8)	0.519	0.187	0.006	1.680	1.163	2.425
Sexual contact with syphilis (control=yes)	19 (5.2)	3 (0.4)			<0.001			
No	275 (74.7)	690 (93.8)	-2.539	0.621	<0.001	0.079	0.023	0.267
Uncertain	74 (20.1)	43 (5.8)	-0.936	0.664	0.159	0.392	0.107	1.442

*Abbreviations: β* partial regression coefficient, *SE* Standard Error, *P* trend significance, *OR* The crude odds ratios, *CI* confidence interval

**Table 4:** Multivariate logistic regression, 2005–2017

Variable	β	SE	Z	P	OR	95%CI	
Razor reuse	0.354	0.176	4.029	0.045	1.425	1.008	2.012
Ear piercing	0.803	0.210	14.586	0.000	2.232	1.478	3.370
Tattoo	1.035	0.448	5.328	0.021	2.814	1.169	6.774
Number of sex partner (control=0)			53.998	<0.0001			
One statilzed sex partner	-0.030	0.325	0.008	0.928	0.971	0.513	1.836
≥2	1.214	0.342	12.611	<0.0001	3.367	1.723	6.580
Condom (control=always)			6.911	0.032			
Occasional	0.057	0.229	0.061	0.805	1.058	0.675	1.658
Never	0.480	0.228	4.419	0.036	1.616	1.033	2.528
Sexual contact with syphilis (control=yes)			42.577	<0.0001			
No	1.454	0.259	31.451	<0.0001	4.282	2.576	7.118
Uncertain	2.160	0.650	11.052	0.001	8.674	2.427	31.001

*Abbreviations: β* partial regression coefficient, *SE* Standard Error, *Z* Chi-square value, *P* trend significance, *OR* The crude odds ratios, *CI* confidence interval

Multiple condition logistic regression model denoted the following risk factors for increasing rates of syphilis infections: multiple sexual partners (OR = 7.1; 95% CI = 3.685, 13.599), razor reuse (OR = 1.7; 95% CI = 1.252, 2.261); ear piercing (OR = 2.7; 95% CI = 1.877, 3.916); tattoo (OR = 3.3; 95% CI =1.492, 7.167); condom occasionally (OR = 2.8; 95% CI = 1.802, 4.399). The estimates of the PAR indicated that number of sex partner for syphilis infection accounted for 22.5% of all TP-positive cases (Table 5). Razor reuse, ear piercing, tattoo, condom, and Sexual contact with syphilis accounted for 14.4, 14.7, 1.8, 12.9, 1.8% respectively (Table 5). However, The estimates of the PAR about uncertain sexual contact with syphilis in TP-positive cases was 8.1%. In fact, 68.78% of all TP-positive cases occurring in subjects were closely related to these risk factor. The PAR was used to assess the importance of a risk factor among subjects because it is a function of both the relative risk of exposure to that factor and the prevalence of exposure within the population.

**Table 5:** Population attributable risk of 4 factors, 2005–2017

Variable	Level	Case	OR	PAR(%)
Razor reuse	0	332	1	
	1	201	1.425	14.4
Ear piercing	0	165	1	
	1	129	2.232	14.7
Tattoo	0	11	1	
	1	17	2.814	1.8
Number of sex partner ≥2	0	231	1	
	1	143	1.616	22.5
Condom (occasional)	0	166	1	
	1	187	3.367	12.9
	0	19	1	
Sexual contact with syphilis	1	3	8.674	1.8
	0	43	1	
Uncertain of sexual contact with syphilis	1	74	4.282	8.1
Total PAR(%)				76.2

*Abbreviations: OR* The crude odds ratios, *PAR* population attributable risk

## Discussion

The safety of transfusions has reached a very high level. Still, some residual risks of TTI remain in consultation before blood donation. The susceptible populations of syphilis is the same as HIV, with similar biological and behavioral factors [19]. A similar situation exists in the infection of HBV and HCV. Risk factors for blood donors infected with syphilis are also risk factors for other blood-borne diseases [20, 21]. In the present study, the rate of TP-positive in primary blood donors was higher than in repeat blood donors among blood donors in Chengdu from 2005 to 2017. WHO noted that HIV can be controlled and facilitated through effective antiretroviral drugs, enabling people living with HIV and those at risk to enjoy a healthy and productive life for a long time [22, 23]. The risk of transmission of HIV is reduced, and the awareness of prevention is weakened, which may be related to the infection of other sexually transmitted diseases.

With the sexual consciousness becoming more and more open, many people use mobile phone software to find sexual partners, which increases the population of multi-sex partners. Multiple sexual partners is a significant risk factor for syphilis infection. In China, the blood donor selection requirements clearly stipulates that multiple sexual partners cannot donate blood [24]. However, there are still some blood donors who deliberately conceal the facts of their multiple sexual partners and enter the blood donation process after filled out health consultation. In the present study, the behavior of multiple sexual partners in the positive group and the control group were 50.8 and 22.6%, respectively. The increase of the proportion of multiple sexual partners in all blood donors have a major impact on blood safety. Different countries adopt different strategies. The UK (excluding Northern Ireland) reduced its blanket ban on MSM (men who had sex with men) donors to a narrower restriction which only prevents MSM from donating blood if they have had sex with other men within the past year [25]. A similar change was made in the U.S. in late 2015 by the FDA [26]. Countries such as Canada and Norway have extended blood donations for six months after replacing a new sexual partner [27–29]. There is a lack of authoritative interpretation of multiple sexual partner concepts in China, especially for the definition of time. The length of the infection window period is an important factor of blood safety, a scientific strategy can ensure blood safety without affecting the supply of blood resources.

The prevailing viewpoint is that syphilis is a sexually transmitted disease. Actually, in our study, shared razors, ear piercings, and tattoos are also the high risk factors for syphilis infection. Sharing cosmetic surgical instrument has been proven to transmit blood-borne diseases. Currently, cosmetic surgery such as tattooing, piercing, rhinoplasty, injection, laser, and liposuction has greatly increased. [30, 31] It has been reported that 17.27% of college students have a history of cosmetic surgery such as rhinoplasty, and 69.4% said they would undergo such cosmetic surgery without considering economic factors [32–34]. Therefore, the blood transmits pathway of syphilis should not be ignored. However, only tattoos and ear piercing have been scheduled to delay be donating blood for one year in China, whereas there is no postponement of blood donation after other cosmetic surgery. The provisions for postponing blood donation after cosmetic surgery should be further improved in future.

Sexual contact with syphilis patients is a highly risk factor for syphilis spread. However, in our survey, many subject are unsure whether their sexual partners were infected with syphilis, the answer between unknown and confirm was no statistically significant in multivariate analysis. The reason may be that syphilis patients do not know whether they have been infected with syphilis. The clinical staging of syphilis infection is different, symptoms are complicated. Moreover, the clinical signs of occult infection are not significant, only serological tests are positive, that may cause misdiagnosis and missed diagnosis. The occult infection accounts for about 50% of the confirmed patients [35, 36]. In addition, the syphilis patients do not recognize the harm of syphilis, or for other reasons, the sexual partner is concealed to her/his sexual partner, and the other partner doesn’t have the knowledge to identify syphilis, which may cause widespread spread of syphilis [37]. In our study, considering of privacy, some respondents were reluctant to inform the research investigator about real situation.

Some of the variables in this study were statistically significant in the univariate analysis (*P* < 0.05), indicating that the variable was a risk factor for syphilis infection, but no statistical significance was found in the multivariate analysis, such as paid sexual, condoms, dental history and acupuncture history. It is maybe relate to the biological activity of *Treponema pallidum* and the size of sample, further research is needed. In this study, paid sexual behavior is also a high risk factor for the spread of syphilis, we included sexual services in the Multiple sexual partners and no longer elaborated. Although the result of marital status was no statistically significant in univariate analysis, in the present study, 9 out of 16 divorced people were TP positive, and nearly half had paid sexual behavior. The cause of such a high rate of syphilis infection remains to be further studied.

Furthermore, screening for high-risk groups before blood donation is now absolutely dependent on pre-donation health consultation. Reporting on medical history and risk behavior by blood donors determines that they can donate blood or need to postpone or withdraw from blood donation. In fact, many blood donors did not seriously fill out the “blood donor health status questionnaire”. On the one hand, it may be that blood donors didn’t understand the contents of the table. On the other hand, some blood donors worried about privacy disclosure when blood was collected on street the open environment. Moreover, the diversity motives of blood donation, also made it impossible for some blood donors to provide true information, therefore, some high-risk groups to enter the blood donation process.

In summary, In order to ensure blood safety and reduce the proportion of blood donation in high-risk groups, it is necessary to strengthen the screening and health survey of blood donors before blood donation. A striking finding of the study is that cosmetic surgery is also one of the risk factors for syphilis infection. Sharing cosmetic surgical instrument which is often overlooked has been proven to transmit blood-borne diseases. Therefore, the blood circulation of syphilis should not be ignored. Moreover, with the rapid development of the economy, the whole country should increase investment in blood collection and supply, and strengthen the promotion of health knowledge. While eliminating high-risk blood donors, it is necessary to ensure that enough low-risk blood donors participate in voluntary blood donation.

## Conclusions

Health consultation and screening of high-risk groups before blood donation need to be further improved. Blood donor recruitment should emphasize on excluding the high-risk donors and recruiting more low-risk blood donors. The seroprevalence of syphilis in blood donors from 2005 to 2017 in Chengdu showed an overall upward trend. However, Chinese MOH permanently defers donors with TP-positive, thus, the rate of TP-positive in primary blood donors was higher than in repeat blood donors. In addition, this study also shows that sharing cosmetic surgical instrument has been proven to transmit blood-borne diseases. Therefore, the syphilis in blood circulation should not be ignored. The research provides a valuable reference for popularizing syphilis-related knowledge and is conducive to reducing the risk behaviors of blood donors.

## Additional file

Additional file 1: Questionnaire of Public health (DOCX 14 kb)

## Data Availability

The datasets used and/or analysed during the current study are available from the corresponding author on reasonable request.

## Abbreviations

CI: Confidence interval
HBV: Hepatitis B virus
HCV: Hepatitis C virus
HIV: Human immunodeficiency virus
MOH: Ministry of Health
MSM: Men who had sex with men
ORs: The crude odds ratios
PAR: Population attributable risk
STD: Sexually transmitted disease
TP: Treponema pallidum
TTIs: Transfusion-transmissible infections
WHO: World Health Organization
