# Supplementary material for: Seroprevalence and risk factors on Syphilis among blood donors in Chengdu, China,from 2005 to 2017
Source: BMC Infect Dis. 2019 Jun 10;19:509. doi: 10.1186/s12879-019-4128-7 (PMC6558839; doi:10.1186/s12879-019-4128-7)
Supplement: Supplementary file 1 — Questionnaire of Public health (DOCX 14 kb) [file 12879_2019_4128_MOESM1_ESM.docx]

Questionnaire of Public health

The contents of this form will be used for public health services and scientific research, and will be kept confidential for those who are not involved in the project. In no case will your profile be quoted and only the overall situation analyzed. The information you provide is very helpful for your personal health and for further improving your health prevention efforts. Based on the spirit of being responsible for science, I hope that you provide the most accurate information possible. Thank you for your cooperation!

Blood donation code**：**__________ Name：________ Tel：_________ Gender：_________ Nationality：___________ Birth：___________ Time of donation：___________

**1**.Home address (or household registration address)：___________

**2**.Marital status？

□ Unmarried □ Married or cohabiting □ Divorce □ Separated □ Widowed

□ Other (Please clarify)____________

**3**.Education?

□ < Primary school □ Middle school□ High school□ Complete university □ Postgraduate

□ Other (Please clarify)____________

**4**.Occupation?

□ Worker □ Farmer □Merchant and commercial service □ Service workers □ Government staff □ Military or police □Medical and health workers □ Student

□ Other (Please clarify)____________

**5**.Have you ever used facials, trims (ears), eyebrows, pedicures, etc. in a beauty salon?？

□Yes □facials □ Pedicures □ Eyebrows □ No □ Never

a.Is the razor you used disposable?

□Usually not disposable □ Sometimes disposable □ Usually disposable □ All disposable

**6**.Have you ever had a cosmetic surgery (such as laser beauty, tattoo/bristle/lip, injection of collagen, microdermabrasion, pierced earrings, etc.,)?

□No □Yes：

If the choice is yes,please answer: how long does the surgery take before your last blood donation?___________

**7**.Have you ever cleaned your teeth, or extracted teeth, or filled a teeth? □ Yes □ No

**8**.Have you ever received acupuncture?

□ No □ Yes：If the choice is yes, please answer: is the needle you use disposable? □Usually not disposable □ Sometimes disposable □ Usually disposable □ All disposable

**9**. Have you ever had a finger prick for blood?

□ No □ Yes □ Unclear

1. Have you received any injections (excluding vaccines)？

□ Accepted ten years ago □ Accepted within ten years □ Often accepted □ Never accepted

**11**. Have you ever injected narcotics with syringes?

□ No □ Yes

If the choice is yes,please answer

a.Do you share syringes or needles for with narcotics others? □ No □ Yes

**12**.Have you ever had a blood transfusion？

□ No □ Yes

If the choice is yes,please answer

1. How many times of blood transfusions have you received? ___________
2. Year of the first blood transfusion: ______ c. Year of the last blood transfusion ________

**13**. How many times have you donated blood?___________

a. Year of the first blood donation：_______ b.Year of the last blood donation:_________

**14**.Whether your skin or mucous membranes have the chance to contact with other's blood or body fluids at work.?

□ No □ Yes

**15**.Number of sexual partners？

□ None □ Have：□ One □ Two or three □ Multiple

**16**.Condom？

□ Always □ Occasional □ Never

**17**. Have you ever provided or received paid sexual services?

□ No □ Yes

**18**.Does any of your sexual partners have had sexually transmitted diseases such as sexually transmitted diseases such as syphilis, AIDS, gonorrhea, genital warts, etc.?

□ No □ Unclear □ Yes____________

**19.** Have you ever lived with someone who has syphilis? □ No □ Unclear □ Yes_________

**20**.Can daily contact with syphilis patients, such as eating and shaking hands, be contagious?

□ No □ Unclear □ Yes
